# The Implementation of Mindfulness-Based Programs in the Swedish Healthcare System – A Survey Study of Service Providers

**DOI:** 10.1177/21649561211049154

**Published:** 2021-11-03

**Authors:** Maria Niemi, Rebecca Crane, Jermo Sinselmeijer, Susanne Andermo

**Affiliations:** 1 Department of Global Public Health, 27106 Karolinska Institutet, Stockholm, Sweden; 2 Centre for Mindfulness Research and Practice, School of Psychology, 1506 Bangor University, Bangor, UK; 3 Department of Global Public Health and Department of Neurobiology Care Sciences and Society, 27106 Karolinska Institutet

**Keywords:** implementation, promoting action on research implementation in health services, mindfulness, mindfulness-based programs, depression, anxiety, intervention integrity, fidelity

## Abstract

**Background:**

The burden of depression and anxiety is on the rise globally. Mindfulness-Based Programs (MBPs) are a particular group of psychosocial programs targeting depression and anxiety. There is growing research and practice interest in MBPs internationally, and they are becoming more commonly implemented in a number of countries’ healthcare services.

**Objective:**

To systematically map the existing provision of MBPs in the Swedish healthcare sector, in order to understand facilitators and barriers to uptake, and so inform future implementation efforts.

**Methods:**

We assessed the experiences of MBP implementation among relevant stakeholders in Swedish healthcare settings through an online survey. The survey was designed to gather data on (1) the evidence-base of practice being implemented; (2) the context in which implementation was taking place and (3) the process of facilitation. Respondents were identified through snowball sampling of key stakeholders.

**Results:**

In total, 129 individuals from 20 of the 21 healthcare regions in Sweden responded to the survey. Our findings showed that there is variation in the types of MBP models being implemented, and that the delivery structure of evidence-based programs were often being modified for implementation. We found some divergence from international guidance on good practice standards for the training of MBP teachers within Swedish implementation processes. The main service context for implementation is primary care; the most important facilitating factors for successful MBP implementation were the presence of a championing individual and support from leadership. The most influential hindering factors for implementation were lack of time, and lack of funding.

**Conclusion:**

To support integrity and fidelity of MBP implementation in Sweden, a strategic plan and good practice guidelines seem necessary. Also, an evidence-based stepped care model for implementation may work to ensure intervention fidelity in cases where time and funding constraints permit.

## Background

Worldwide, the burden of mental disorders is on the rise, and depression has become a leading cause of disability. By 2017, 264 million people were affected with either chronic depression or major depressive disorder.^1,2^ The World Health Organization’s Mental Health Action Plan 2013–2020 calls for research and implementation of strategies for prevention in this area.^3^ In Sweden, Common Mental Disorders (CMDs), including depression and anxiety, account for approximately 90% of all sick leave due to mental illness.^4,5^ Adults with depression and generalized anxiety are mainly treated with antidepressant medication and psychosocial interventions. This paper focuses on the implementation of one particular group of evidenced based psychosocial interventions targeting CMDs – Mindfulness-Based Programs (MBPs).

Mindfulness has been described as ‘the awareness that arises from paying attention, on purpose, in the present moment and non-judgmentally’.^6^ In the last decades, the number of scientific studies on MBPs has increased exponentially, and their use has spread in clinical practice as well as in society at large. Mindfulness-Based Stress Reduction (MBSR)^7^ and Mindfulness-Based Cognitive Therapy (MBCT)^8^ are the two most common programs offered in clinical settings, and the ones that have been most extensively researched.^9^ According to a recent meta-analysis, MBPs have the strongest evidence for managing depression.^10^ Mindfulness-Based Cognitive Therapy significantly reduces the risk of relapse in recurrent major depressive disorder, being most effective for participants with three or more previous episodes and at least as effective as maintenance antidepressant medication.^11,12^ Consistent with these findings, another meta-analysis concludes that MBCT and MBSR are effective for a variety of psychological problems, especially for reducing anxiety, depression, and stress.^13^ However, in parallel with increasing research evidence of the benefits of MBPs, reports of potential harm from mindfulness practice have recently emerged. A recent meta-analysis of adverse events during and after meditation – including mindfulness – concluded that such events are not uncommon and may occur even in individuals without previous mental health problems.^14^ A recent conceptual paper recommended that research should more specifically address factors that may contribute to the risk of adverse events from MBPs, including program-, participant- and teacher-related factors.^15^

### Implementation of MBPs

There is growing interest in MBPs globally, and they are becoming more commonly implemented in a number of countries’ healthcare services.^16,17^ A review of the literature on implementation of MBPs internationally reveals that this research area has not yet been extensively addressed. Indeed, a mapping published in 2015 of the MBP evidence offered a framework for understanding the strengths and vulnerabilities of the current research on MBSR and MBCT.^9^ Seven recommendations were made to ensure that the forward trajectory of MBP research developments is strong – two of these relate to the importance of future research engaging with questions related to the practical implementation of MBPs. One of these recommendations focused on the question of clinician as well as quality and integrity of the delivery of MBPs. The second recommendation related to the risk of developing ‘orphan innovations’, which may contribute to the loss of intervention effectiveness that often occurs in real-world clinical settings, due to various reasons. There are some studies on the implementation of MBPs in schools,^18,19^ and a smaller number on the implementation of MBPs in healthcare systems.^20-22^ This paper reports on a study investigating the MBP implementation challenge in the Swedish healthcare system.

The ASPIRE study on the implementation of MBPs’ in the UK health service is the most thorough investigation of the factors influencing implementation to date. The findings point to four factors for successful implementation, including (1) careful adaptation of MBCT services to ensure it fits within the local context, by integrating it with existing care pathways, organizational structures and service priorities; (2) drawing on a diversity of evidence to support the drive to implement (i.e., including but going beyond scientific evidence by embracing evidence that emerges from local service evaluation, first-person accounts and participant preferences); (3) the presence of MBCT implementers, or so-called ‘champions’, who have key leadership or influential roles within the relevant context for implementation, and (4) ‘pivot points’ along the implementation pathway that provided windows of challenge or opportunity (i.e., the implementation champions were skilled in harnessing opportunities created by issues such as service reorganizations or change of leadership to accelerate the implementation endeavor).^23^

Recently, MBCT and MBSR have been included in the depression treatment guidelines of the Swedish National Board of Health and Welfare.^24^ However, even if a psychosocial intervention has a vast evidence-base and is recommended in national treatment guidelines, its value in reality is determined by how widely available it is in the health service.^25^ Even in the United Kingdom, where it has been national policy since 2004 to embed MBCT in health service delivery, there is a high variability in the access to MBPs.^20^ In the Swedish context, there is a lack of systematized knowledge regarding the provision of MBPs – including the types of programs being provided, the level of training among MBP teachers, and the extent to which programs are available. Also, Swedish guidelines for mental health promotion in the workplace point out that there is a lack of systematized certification procedures and competence criteria for the selection and assessment of MBP teachers in Sweden.^26^ Therefore, a systematic approach to investigating the process of MBP implementation in Sweden may inform and optimize the delivery approach going forward.

The present study is based on the implementation framework ‘Promoting Action on Research Implementation in Health Services’ (PARIHS).^27^ This framework is utilized in health service research and practice to guide implementation, with a focus on understanding the complexities of the transition from evidence to practice. Translation from evidence to practice is generally not a straight-forward process. Practice often lags behind what is known to be evidence-based best practice. In the PARIHS framework, successful implementation is represented as an interaction between (1) the nature and the type of *evidence* being implemented; (2) the qualities of the *context* in which it is being implemented and (3) the process of *facilitation*. The PARIHS framework utilizes a broad perspective on *evidence*, including rigorous qualitative and quantitative research, practice-based clinical evidence, and patient experiences and preferences. The term *context* in the framework refers to the setting within which evidence is transferred into practice. Important aspects in this process include culture, leadership and the continuous evaluation of the process of implementation. Finally, the term *facilitation* focuses on the process of facilitating evidence into practice. This can, for example, occur through the activities of a key stakeholder with the right knowledge and skills to help others in the process of implementation. Altogether, the framework can be used to map factors that are important in enabling successful implementation before, during and after the efforts.^27^

The present study has also been informed by the ASPIRE study (see above), which also utilized the PARIHS framework to develop an explanation for MBCT implementation in the UK health service. The aim of the present study is to systematically map the existing provision of MBPs in the Swedish healthcare sector, informed both by the PARIHS framework and the ASPIRE study. Our aim is that the resulting understanding will inform the next steps in the implementation journey both in Sweden and internationally.

## Methods

We assessed experiences of MBP implementation among relevant stakeholders in Swedish healthcare settings. An online survey, based on the PARIHS framework had previously been developed and used in the UK.^25^ This was designed to gather data on (1) the evidence-base of practice being implemented; (2) the context in which the implementation was taking place and (3) the process of facilitation. We utilized the survey previously used in the UK study^25^ as a base for the present survey, added some modifications to that survey based on findings from the ASPIRE project,^23^ and included some more specific questions regarding facilitating and hindering factors for implementation in the Swedish context. The structured web survey consisted of questions with closed answer options as well as open questions allowing participants to elaborate their responses (see a translated version of the survey in the Supplemental Material). The survey scoped existing provision and focused on perceptions about MBPs, ascertained views about embedding MBPs into service delivery, including models of teacher training, facilitators, barriers, costs and benefits. Respondents were identified through snowball sampling with key stakeholders. We aimed to include stakeholders from the following groups, in order to provide a broad and nuanced view of MBP implementation: MBP teachers, primary and mental healthcare service managers, commissioners, referrers, regional and county coordinators, and MBP teacher trainers.

### Sampling

The survey was e-mailed out to respondents during autumn 2019. We aimed to include commissioners, managers, referrers and service coordinators at regional and county levels as respondents in the study. However, it was challenging to obtain survey responses from other stakeholders than MBP providers and teacher trainers (those who could be regarded as ‘champions’ of MBP provision), despite attempts to reach them via targeted e-mails. Presumably, busy working schedules and time priorities render those more specifically interested in advancing MBP provision more likely to respond to the survey, and others less likely to do so. Therefore, the study findings do not reflect the views of the broader range of stakeholders that we aimed for, but rather mainly of MBP providers and teacher trainers. There are two main providers of MBP teacher training in Sweden**:** Center for Mindfulness Sweden, providing teacher training in MBSR, and Mindfulnesscenter, providing teacher training in an MBP that is a modified version of MBSR, called the Here & Now program.^28^ The survey was disseminated though the networks of both of these centers.

Our aim was to include participants from all 21 regions in Sweden, in order to gain a broad geographical perspective on MBP implementation in the country. Within each area, we aimed to begin with a stakeholder who had knowledge of MBP service delivery across their region, and then to snowball out to stakeholders who were involved in the delivery of MBP services, commissioning of the service, use the service or referral to the service. The study had ethical approval from the Swedish Ethical Review Board, approval number 2019-02952.

## Statistical Analysis

The software used for analysis was IBM SPSS version 26. Descriptive statistics were used to describe the frequencies of the baseline variable categories among the participants as well as other survey responses.

## Results

The findings from this survey provide a perspective from MBP teachers and teacher trainers on if and how MBPs are being delivered across Sweden, including the factors that have facilitated and/or hindered its implementation both at the level of commissioning and service delivery. In total, 129 individuals from 20 of the 21 geographical regions in Sweden responded to the survey. The following section reporting the results of the survey is structured by the PARIHS framework: (1) the nature and the type of *evidence* being implemented; (2) the qualities of the *context* in which it is being implemented; and (3) the process of *facilitation*. For context, prior to presenting the results we summarize the training routes available for MBP teachers in Sweden – see Supplemental Material.

1. The evidence-base of practice

We found that there was broad variation in the types of MBP models that were being implemented (see Tables 1 and 2). Some were implementing the well-established and evidenced programs of MBCT and MBSR and delivering with fidelity to the curriculum guides; some were implementing the Here & Now program, which has preliminary empirical support,^28^ and an ongoing commitment to develop its evidence base; whilst, many others were implementing programs that were variations and modifications of the original programs which do not have empirical support.

**Table 1.** Types of MBP being implemented – total and per service.

Manual	n	% of n	Primary care	Occupational healthcare	Psychiatric care	Hearing care	Youth health center	Other specialist care	Other
MBSR 2017	19	14.7	3	0	2	4	2	1	7
MBCT 2013	8	6.2	4	3	1	0	0	0	0
Here & Now 2014	43	33.3	27	5	2	1	1	2	5
Modif. Here & Now	9	7.0	5	1	1	0	1	1	1
Own adaptation	12	9.3	5	0	1	0	2	2	0
Unknown	2	1.6	1	0	0	0	0	0	1

MBP, mindfulness-based programs; MBSR, mindfulness-based stress reduction; MBST, mindfulness-based cognitive therapy.

**Table 2.** Weekly meetings and home practice.

N of weekly meetings	N	% of N
Less than 6	15	11.6
6–8	26	20.2
8–10	41	31.8
More than 10	6	4.7
Unknown	5	3.9
Weekly meeting length (hrs)		
<1r	7	5.4
1–1.5	36	27.9
1.5–2.5	40	31.0
>2.5	7	5.4
Unknown	3	2.3
Assigned home practice/day		
None	5	3.9
<10 mins	10	7.8
10–20 mins	33	25.6
20–45 mins	27	20.9
≥45 mins	11	8.5
Unknown	6	4.7

There was a lot of divergence from international norms of good practice in MBP provision. The most common minimum criteria for MBP providers that were required by the work settings was that they have a professional license to practice, and have taken the ‘Here & Now’ stage 1 training. These minimum criteria were in place in 28.7% of the cases (n = 37). The second most commonly implemented minimum criterion in place was having a license to practice as well as a full MBSR or MBCT teacher training (11.5%; n = 15). Other work settings had other forms of minimum criterion, or none at all. As regards the level of fidelity to the form of the manualized MBP programs that were implemented (detailed in Supplemental Material), information regarding the number of weekly meetings, the weekly meeting length and the assigned home practice is given in Table 2.

2. The context of implementation

Table 3 presents the geographical spread, professional training and service context of the survey participants.

**Table 3.** Survey participants; N = 129.

Region (total population in region)	Freq	% of N
Blekinge (160 k)	6	4.7
Dalarna (287 k)	3	2.3
Gotland (59 k)	1	0.8
Gävleborg (287 k)	4	3.1
Halland (330 k)	5	3.9
Jämtland (131 k)	2	1.6
Jönköping (362 k)	6	4.7
Kalmar (245 k)	3	2.3
Kronoberg (200 k)	2	1.6
Norrbotten (251 k)	4	3.1
Skåne (1366 k)	32	24.8
Stockholm (2353 k)	26	20.2
Södermanland (295 k)	4	3.1
Uppsala (378 k)	1	0.8
Värmland (282 k)	0	0
Västerbotten (271 k)	3	2.3
Västernorrland (245 k)	4	3.1
Västmanland (275 k)	2	1.6
V. Götaland (1714 k)	15	11.6
Örebro (303 k)	2	1.6
Östergötaland (462 k)	4	3.1
**Occupation**	Freq	% of N
Occupational therapist	6	4.7
Psychotherapist	7	5.4
Medical doctor	11	8.5
Counselor	31	24.0
Nurse	13	10.1
Physiotherapist	25	19.4
Psychologist	12	9.3
Midwife	4	3.1
Stage 1 psychotherapist	12	9.3
Other	8	6.2
**Workplace**	Freq	% of N
Primary care	60	46.5
Psychiatry	11	8.5
Occupational healthcare	10	7.8
Hearing care	5	3.9
Pain and burnout clinic	6	4.7
Rehabilitation	8	6.2
Youth care center	9	7.0
Hospital specialist care	11	8.5
Private practice	6	4.7
Maternal health care	3	2.3

Among those who responded to the survey, 27.9% (N = 36) indicated that MBPs had not been implemented in their workplace. The rest of the respondents indicated that MBPs were actively being implemented, but with some variation. The most common response (35.7%; N = 46) was that MBPs had been implemented with success, and with good support from the organizational leadership. The rest of those who responded reported that MBPs had been implemented, but with some difficulty: either the MBP did not play a significant role in the workplace and was not supported by leadership (12.4%; N = 16); or implementation was supported by the organization, but funding was an issue or difficulty (24%; N = 31).

The patient groups that MBPs were being offered to were as follows: Anxiety (56.6%, n = 73); stress (57.4%; n = 74); mild depression (51.2%; n = 66); prevention of depression relapse (34.9%; n = 45); pain (32.6%; n = 42) and ‘other’, including trauma, tinnitus, fatigue, burnout, fear of childbirth, sleeping problems, ADHD, and bipolar disorder (32.6%; n = 42).

3. The process of facilitation

Important areas of facilitating and hindering factors to implementation were illustrated through the survey responses as follows. Among the respondents, the majority (49.6%; n = 64) thought that there was either no support or not very much support from leadership in their organization for the implementation of MBPs. Among those who agreed to the statement that leadership was supportive of implementation, 38.0% (n = 49) somewhat agreed, and 8.5% (n = 11) agreed fully. The majority of participants expressed that there were no opportunities (15.5%; n = 20), or very little opportunities (20.9%; n = 27) for professional development and supervision for MBP providers, while others indicated that such opportunities were provided either in part (4.1%; n = 53) or fully (18.6%; n = 24). The majority of participants were allowed to use their working hours for MBP provision and preparation (41.1% agreed in part, 18.6% agreed fully); while some indicated that they could not fit this work within their working hours (20.9% agreed in part; 15.5% agreed fully). Administrative support was obtained by the majority, where 39.5% agreed in part and 15.5% agreed fully; while some also indicated that they did not receive administrative support for MBP implementation (27.9% agreed in part, 13.2% agreed fully). Finally, respondents were asked to tick boxes to indicate which facilitating and hindering factors for implementation were relevant for their setting – the results from the responses are summarized in Table 4.

**Table 4:** Frequency of experienced facilitating and hindering factors for MBP implementation; N = 129.

Facilitating/hindering factor	n	% of N
Presence of a ‘champion’ individual	90	69.8
Support from leadership	77	59.7
Adequate funding	33	25.6
Administrative support	14	10.9
Lack of teacher competence	28	21.7
Lack of time	59	45.7
Lack of funding	40	31.0
Organizational changes/disruption	26	20.2
Other	21	16.3

## Discussion

Here, we examine the survey findings within the structure of the PARIHS framework: *evidence, context* and *facilitation*.

### Evidence

The findings revealed that there was broad variation in the types of programs being implemented, and that the level of fidelity to evidence-base programs such as MBSR and MBCT was frequently low, with modifications such as the length and number of sessions and the amount of home practice being assigned. Although the programs that were being implemented draw on the wider evidence for MBPs, the specific adapted programs do not have research support. The exception to this is the Here & Now program which is a Swedish adaptation of MBSR that has preliminary evidence,^28^ and a commitment to developing the evidence going forward.

Ensuring a good ‘fit’ between the program and the delivery context was a key recommendation from the UK ASPIRE research. This process involves the alignment between the intervention and context, including efforts to make the intervention fit with existing local service strategies, priorities, pathways, resources and the ethos or culture of the service. Also, a good fit requires recognition of national and service performance targets and alignment with managers’ and service users’ needs and interests.^22^ These factors were all issues that the Swedish implementers were facing. Implementers are navigating the tension between delivering evidenced-based interventions with high fidelity within organizational settings that are often not able or willing to release the level of resources needed for gold standard delivery vs reducing the ‘dose’ of the intervention and therefore, the resource demand to overcome organizational barriers.

One helpful way of approaching the challenge of the tension of the benefits of high fidelity/high resource interventions vs lower dose/lower resource interventions is a ‘stepped care’ implementation model.^20^ A stepped care model for implementing mindfulness might involve a number of tiers depending on symptom severity.

Indeed, from our findings, it appears that the beginnings of a stepped care approach are being implemented, with many shortened and adapted versions of evidence-based programs being in place. Also, other researchers have suggested additional ways in which MBPs could be modified to fit context, including open or rolling admission groups where both newcomers and prior attendees can attend any treatment session, or by shortening treatment sessions.^21^ However, to date there is not enough evidence to guide the implementation of MBP in stepped care formats, rolling admissions or shorter sessions. In the Swedish context, there appears to be a lack of provision of the full evidence-based programs MBSR and MBCT as recommended in the national guidelines.^23^ Also, from the results of this survey, it is unclear whether the modifications of the programs have been conducted in response to patient needs, or whether such modifications are instead guided by situational and contextual factors unrelated to patient needs.

From our findings, it appears clear that training of MBP teachers overall is not in line with international guidelines for good practice established by a consensus of trainers working globally (International Mindfulness Integrity network^29^). These guidelines include attendance at silent mindfulness meditation retreats as part of the teacher training – something that is not included in for example the Here & Now stage 1 training, which we found to be most broadly implemented. Our finding here again mirrors those from the ASPIRE study, where teacher training levels could vary from simply reading the MBCT manual,^30^ to having completed full training pathways.^23^ Indeed, Crane and Kuyken have based on their findings in the UK, detailed some key aspects to ensuring the quality of professional MBP training, and these include (a) the content, method and process of MBP teacher development and training; (b) good practice standards and (c) the definition of skills needed to teach mindfulness groups.^25^ These aspects seem not to be systematically in place in the clinical settings in Sweden that were studied through the present survey. However, good practice guidelines are in place in the UK,^31^ for instance, and they could provide a basis to delineate more clearly what would be best practices for the Swedish context.

### Context

The survey participants worked mainly in the Skåne, Stockholm and Väster Götaland regions, and this can be expected since these three regions are the most populous ones in Sweden. The main professional backgrounds of MBP providers were counselor, physiotherapist, followed by nurse, psychologist and ‘stage-1’ psychotherapist (in Sweden, psychotherapist training for non-psychologists is completed in two stages, and the stage-2 training leads to a psychotherapy license). This pattern is somewhat different from the one described by Crane and Kuyken in the UK,^25^ where psychologists were the main group administrating MBPs, followed by occupational therapists, social workers and nurses specializing in psychiatry.^25^

Mindfulness-Based Programs were being implemented in a broad range of services beyond psychiatry and primary health care from depression and anxiety as suggested by the national guidelines. The main service of implementation was primary care, and this is indeed in line with suggestions made by Demarzo et al^20^ of MBP implementation, due to primary care being an accessible gateway into the healthcare system. Additional services where implementation was taking place ranged from hearing care, maternal health care, youth care centers and pain and rehabilitation clinics. The implementation of MBPs for some of these patient groups is not fully in line with what is recommended in the national guidelines or supported by research evidence for MBPs. Therefore, our findings indicate that the implementation of MBPs in Sweden may in some places be rushing ahead of the evidence – in similar ways that have been discussed in international research.^32^

### Facilitation

In line with the ASPIRE findings,^23^ the main facilitating factor for MBP implementation indicated by our survey participants was the presence of a championing individual. The ASPIRE findings elucidate that some particular characteristics of these champion individuals are particularly important. These include status within the organizational hierarchies as well as social skills, and they were often self-designated individuals who ‘championed’ grass-roots implementation.^23^ Our findings in the present study do not provide detail about the particular characteristics of these championing individuals that are deemed important in the Swedish settings. This would be a useful line of inquiry for the future. The second most important facilitating factor indicated by our participants was support from leadership. In terms of hindering factors for implementation, lack of time was indicated most frequently, followed by lack of funding.

## Limitations and Strengths

A strength of this study was that it spanned all but one region in Sweden, thus providing a relatively good geographical representation of the whole country. Also, the respondents worked in various forms of healthcare services, thus providing a broad picture of the various implementation settings. A clear limitation, however, is that the survey responses came mainly from MBP providers, that is, so-called ‘champions’. In order to gain a clearer and fuller picture of the facilitating and hindering factors for implementation, the view of service managers, coordinators and referrers is also needed. We did aim to survey these groups of respondents but did not receive any responses despite e-mail reminders. Presumably, busy working schedules and time priorities render those more specifically interested in advancing MBP provision more likely to respond to the survey, and others less likely to do so.

## Conclusion and Implications

As of yet, in Sweden, MBP strategy appears to be in place only at the national level in the form of guidelines. Our study did not show evidence of the existence of more regional or local strategies, as the actual implementation appears patchy and versatile. Our findings indicate that MBPs are being implemented in various healthcare contexts and modified in various ways, which raises questions about intervention fidelity and integrity. Also, future research that addresses the question of ‘dose-response’, for example, for patients with less severe symptoms, as shorter or less intensive forms of MBPs may be more suitable for broad implementation in a number of settings. Since time and funding are indicated as main hindrances for implementation, the development of evidence-based stepped care models may ensure high quality implementation for those patients who would most benefit from MBPs, while patients with less severe symptoms could benefit from ‘lighter touch’ programs. In summary, taking the long view and setting good practice guidelines for MBPs seems to be pivotal for good quality implementation of MBPs in Swedish healthcare settings.

## Supplemental Material

sj-pdf-1-gam-10.1177_21649561211049154 – Supplemental Material for The Implementation of Mindfulness-Based Programs in the Swedish Healthcare System – A Survey Study of Service Providers Click here for additional data file. Supplemental Material, sj-pdf-1-gam-10.1177_21649561211049154 for The Implementation of Mindfulness-Based Programs in the Swedish Healthcare System – A Survey Study of Service Providers by Maria Niemi, Rebecca Crane, Jermo Sinselmeijer and Susanne Andermo in Global Advances in Health and Medicine
