# Supplementary material for: The Implementation of Mindfulness-Based Programs in the Swedish Healthcare System – A Survey Study of Service Providers
Source: Glob Adv Health Med. 2021 Nov 3;10:21649561211049154. doi: 10.1177/21649561211049154 (PMC8573620; doi:10.1177/21649561211049154)
Supplement: sj-pdf-1-gam-10.1177_21649561211049154 – Supplemental Material for The Implementation of Mindfulness-Based Programs in the Swedish Healthcare System – A Survey Study of Service Providers [file sj-pdf-1-gam-10.1177_21649561211049154.pdf]

Appendix 1:

Supplemental material for "The implementation of Mindfulness Based Programs in the Swedish healthcare system – a survey study of service providers"

Research questionnaire file

by Maria Niemi, Rebecca Crane, Jermo Sinselmeijer and Susanne Andermo in

"Global Advances in Health and Medicine"

This questionnaire is designed to take no more than 30-45 mins to complete. We are currently only surveying the implementation of Mindfulness-Based Interventions (MBIs) within the Swedish Health Service. Please fill this in in relation to provision of MBI services in your local area.

Q1. Which region do you work in?

__ Blekinge
__ Dalarna
__ Gotland
__ Gävleborg
__ Halland
__ Jämtland
__ Jönköping
__ Kalmar
__ Kronoberg
__ Norrbotten
__ Skåne
__ Stockholm
__ Södermanland
__ Uppsala
__ Värmland
__ Västerbotten
__ Västernorrland
__ Västmanland
__ Väster Götaland
__ Örebro
__ Östergötland

Q2. Which health service organization do you work in?

Specify:

Q3. What is your role in the health service that you work in (profession and employment)

Specify:

Based on the following definition of MBIs, please answer the questions below regarding MBI service delivery:

MBI:s are programmes that are informed by a clear rationale; teacher-led; have been developed to be scaleable; have a set curriculum, typically at least eight sessions with 30 – 45 mins daily mindfulness home practice, incremental development and experiential learning; and have a clear commitment to be evidence-based. The following courses comply to this definition:

Mindfulness Based Stress Reduction (MBSR)
Mindfulness Based Cognitive Therapy (MBCT)
Breathworks
Mindfulness Based Living Course (MBLC)
Mindfulness Based Childbirth and Parenting (MBCP)
Mindfulness Based Relapse Prevention (MBRP)
Here & Now program (Här & Nu, based on MBCT but with shorter weekly meetings and home practices)

Q4. Below are four descriptions of MBI services. These are broad descriptions and may not match local circumstances perfectly. Nevertheless, which description most closely matches your views about MBI services in your organisation? If you work in private practice answer with respect your geographical region. Please tick one of the boxes (A, B, C or D).

- ☐ ***A.*** *There are no MBI classes available within this organisation.*

- ☐ ***B.*** *We do have MBI services here, but they do not play a major role. There are some interested clinicians who are delivering classes but there is little organisational support for developing the service.*

- ☐ ***C.*** *The MBI services in this organisation are well supported by clinical staff but we are struggling to manage with the available resources. Services are slowly improving but there is a long way to go. With sufficient resources, we could really develop MBI services*

- ☐ ***D.*** *The MBI services in this organisation are thriving. We are actively supported by management and by clinical staff on the ground and see the development as successful and innovative.*

Q5. If you answered B, C or D, please specify what MBI is being provided and answer the questions a, b and c below

---

Q6. Please respond to the following questions in relation to your response in Q5, regarding which MBI is being provided:

Number of sessions:

- ☐ *Less than 6*
- ☐ *6-8*
- ☐ *8-10*
- ☐ *More than 10*

Length of sessions:

- ☐ *Less than an hour*
- ☐ *1-1.5 hours*
- ☐ *1.5-2.5 hours*
- ☐ *More than 2.5 hours*

Home practice:

- ☐ *No home practice*
- ☐ *Less than 10 minutes a day*
- ☐ *10-20 minutes a day*
- ☐ *20-45 minutes per day*
- ☐ *More than 45 minutes per day*
- ☐ *I don’t know*

Q8. In relation to your answer to Q5, which manual or guide is being followed?

- ☐ *MBSR manual from 2017*
- ☐ *MBCT manual from 2013*
- ☐ *Här & Nu manual from 2011*
- ☐ Other - please specify:

____

____

Q9. Do you deliver any interventions that draw on mindfulness, outside of the definition in Q.5?

Please specify:

____

____

Q10. This question is looking for your views about the obstacles and facilitators to MBI implementation within your organisation. For each statement, please tick the box that matches most closely your views.

a. There is financial support for training clinicians to deliver MBI

- ☐ *strongly agree*
- ☐ *agree*
- ☐ *disagree*
- ☐ *strongly disagree*

b. There is support for MBI teachers to maintain good practice for delivering classes (e.g. supervision, attendance on retreats, continued professional development opportunities)

- ☐ *strongly agree*
- ☐ *agree*
- ☐ *disagree*

- ☐ *strongly disagree*

c. Trained MBI teachers are given managerial support to prepare for classes and to deliver classes within their working hours

- ☐ *strongly agree*
- ☐ *agree*
- ☐ *disagree*
- ☐ *strongly disagree*

d. There is a fit for purpose room available to offer MBI classes in the organisation

- ☐ *strongly agree*
- ☐ *agree*
- ☐ *disagree*
- ☐ *strongly disagree*

e. There is an appropriate level of available administrative support for setting up and running MBI classes

- ☐ *strongly agree*
- ☐ *agree*
- ☐ *disagree*
- ☐ *strongly disagree*

f. It is easy to get sufficient and appropriate referrals to MBI classes

- ☐ *strongly agree*
- ☐ *agree*
- ☐ *disagree*
- ☐ *strongly disagree*

g. The clinicians who refer to the classes have a clear understanding of the intentions of MBI

- ☐ *strongly agree*
- ☐ *agree*
- ☐ *disagree*
- ☐ *strongly disagree*

Q11. Does the organisation you work within have a strategic plan to develop the implementation of MBI services?

- ☐ Yes
- ☐ No
- ☐ There is no strategy at present, but we are working on developing one.

If yes please summarise the aims of the strategy

____

____

Q12. This section is only relevant if your organisation is already implementing MBI services. If this is not the case for you please go to Q. 13 below.

a. To which client group(s) are MBI classes offered in your organisation? *(tick all that apply)*

- ☐ Vulnerable to recurrent depression, currently in remission
- ☐ Recurrent anxiety
- ☐ Residual depression
- ☐ Adjustment disorder/stress
- ☐ Chronic pain
- ☐ Other – please sepcify

____

____

b. How many MBI courses were offered in your organisation in the last 12 months?

- ☐ 1
- ☐ 2
- ☐ 3
- ☐ 4 or more – please specify amount

c. How many individuals have taken an MBI course within your organisation in the last 12 months?

- ☐ 0-5
- ☐ 6-10
- ☐ 11-15
- ☐ 16-20
- ☐ 21-25
- ☐ 25-30
- ☐ 30+

e. Please estimate the number of clinical hours required to deliver one 8-session MBI class including class contact time, room set up, session preparation, assessment/orientation, individual participant support between sessions

- ☐ 20 - 30
- ☐ 30 - 40
- ☐ 40-50
- ☐ 50 – 60

Any further comments?____

____

f. If you are employed to deliver MBI classes, how many hours are you employed to delivery one 8-session MBI class?

- ☐ Less than 20
- ☐ 20 - 30
- ☐ 30 - 40
- ☐ 40-50
- ☐ 50 – 60
- ☐ I am not employed to deliver MBI classes

Comment: ____

____

g. Which mindfulness practice recorded materials do you give your participants

- ☐ I have recorded my own
- ☐ Camilla Sköld's
- ☐ Ola Schenström's
- ☐ Other – please specify

____

h. Which professionals within your organisation teach MBI (tick all that apply)

- ☐ Clinical psychologist
- ☐ Occupational therapist
- ☐ Social worker
- ☐ Nurse
- ☐ Medical Doctor
- ☐ Physical therapist

- ☐ Other – please specify

____

i. Formal evaluation of outcomes of MBI (standardised measures and/or written feedback) participants is routinely undertaken

- ☐ *strongly agree*
- ☐ *agree*
- ☐ *disagree*
- ☐ *strongly disagree*

Please describe how:____

____

j. When deciding who is suitable for MBI classes, what are your inclusion and exclusion criteria?

Inclusion criteria____

____

Exclusion criteria____

____

k. Do you offer follow-ups and/or reunions for people who have been through your MBI classes?

- ☐ *no*
- ☐ *yes*

If yes how do you do this? _____________________________________

____

l. Does your organisation have minimum training criteria for MBI providers?

- ☐ *no*
- ☐ *yes*

m. Within your organisation what are the minimum training requirements to offer MBI classes?

- ☐ *professional training in a core profession only*
- ☐ *professional training in a core profession and some MBI training (e.g. one day workshops)*
- ☐ *professional training in a core profession and Here & Now program stage 1 training*
- ☐ *professional training in a core profession and Here & Now program stage 2 training*
- ☐ *professional training in a core profession and completion of the full MBSR or MBCT instructor training pathway as provided by Center for Mindfulness Sweden (Camilla Sköld) or other equivalent abroad.*

Other – please specify: ____

____

n. What level of supervision do you use when are running MBI classes?

- ☐ *none*
- ☐ *peer group supervision monthly/fortnightly/weekly* **(circle one)**
- ☐ *one to one supervision with a more experience MBI therapist monthly/fortnightly/weekly* **(circle one)**

Comment: ____

____

o. Within which service(s) are MBI groups cited within your organisation (tick any that apply):

- ☐ primary care
- ☐ psychiatric outpatient services
- ☐ secondary care
- ☐ Other – please specify____

Q13. According to your experience, which are the main facilitating factors (A) and hindering factors (B) for MBI implementation in your organisation?

- ☐ A1: The presence of (a) champion individual(s) within the organisation
- ☐ A2: Support from leadership
- ☐ A3: Funding
- ☐ A4: Administrative support
- ☐ B1: lack of relevant competence/education
- ☐ B2: lack of time
- ☐ B3: Lack of funding
- ☐ B4: Organisational changes
- ☐ Other – please specify_____________________________________

Q.14 Are there any comments you would like to add about MBI services or about any of the issues raised by this questionnaire? For example, how would you like to see MBI services develop in the future?

____

____

____

____

____

Thank you very much for your time.

- ☐ Please tick this box if you would be willing to be contacted for an in-depth interview or would like to recommend someone else in your organization to be contacted for an interview on this topic.

If you ticked the box above, please provide your contact details or the contact details of the person who you recommend for an interview:

Name:

Address:

e-mail address:
